# Vick's Floral Guide for 1880

**Published:** 1880-01

**Authors:** 


*Vick's Floral Guide for* 1880. We are in receipt of another beautiful volume of this enterprising florist for the new year, illustrated by handsome colored chromos of everlasting flowers and sweet peas. The seeds, flower and vegetable of this celebrated grower, are well known throughout the entire country, and give satisfactory results to all planting them. In the Floral Guide much valuable information can be obtained, and the illustrations are profuse and instructive. Address James Vick, Rochester, New York.

